# What do healthcare professionals need to turn risk models for type 2 diabetes into usable computerized clinical decision support systems? Lessons learned from the MOSAIC project

**DOI:** 10.1186/s12911-019-0887-8

**Published:** 2019-08-16

**Authors:** Giuseppe Fico, Liss Hernanzez, Jorge Cancela, Arianna Dagliati, Lucia Sacchi, Antonio Martinez-Millana, Jorge Posada, Lidia Manero, Jose Verdú, Andrea Facchinetti, Manuel Ottaviano, Konstantia Zarkogianni, Konstantina Nikita, Leif Groop, Rafael Gabriel-Sanchez, Luca Chiovato, Vicente Traver, Juan Francisco Merino-Torres, Claudio Cobelli, Riccardo Bellazzi, Maria Teresa Arredondo

**Affiliations:** 1 Universidad Politécnica de Madrid, Madrid, Spain; 2 University of Manchester, Manchester, UK; 3 University of Pavia, Pavia, Italy; 4 Universidad Politécnica de Valencia, Valencia, Spain; 5 Medtronic Ibérica, Madrid, Spain; 6 University of Padova, Padua, Italy; 7 Asociación Española para el Desarrollo de la Epidemiología Clínica, Madrid, Spain; 8 National Technical University of Athens, Athens, Greece; 9 Lund University Diabetes Centre, Malmö, Sweden; 10 Istituti Clinico Scientifici Maugeri Hospital of Pavia, Pavia, Italy; 11 Hospital La Fe, Valencia, Spain

**Keywords:** Type 2 diabetes, Computerized decision support systems, Risk modelling, Human centred design, Multi-disciplinary approach

## Abstract

**Background:**

To understand user needs, system requirements and organizational conditions towards successful design and adoption of Clinical Decision Support Systems for Type 2 Diabetes (T2D) care built on top of computerized risk models.

**Methods:**

The holistic and evidence-based *CEHRES Roadmap*, used to create eHealth solutions through participatory development approach, persuasive design techniques and business modelling, was adopted in the MOSAIC project to define the sequence of multidisciplinary methods organized in three phases, *user needs*, *implementation* and *evaluation*. The research was qualitative, the total number of participants was ninety, about five-seventeen involved in each round of experiment.

**Results:**

Prediction models for the onset of T2D are built on clinical studies, while for T2D care are derived from healthcare registries. Accordingly, two set of DSSs were defined: the first, *T2D Screening*, introduces a novel routine; in the second case, *T2D Care*, DSSs can support managers at population level, and daily practitioners at individual level. In the *user needs phase*, *T2D Screening* and solution *T2D Care at population level* share similar priorities, as both deal with risk-stratification. End-users of *T2D Screening* and solution *T2D Care at individual level* prioritize easiness of use and satisfaction, while managers prefer the tools to be available every time and everywhere. In the *implementation phase*, three Use Cases were defined for *T2D Screening*, adapting the tool to different settings and granularity of information. Two Use Cases were defined around solutions *T2D Care at population* and *T2D Care at individual*, to be used in primary or secondary care. Suitable filtering options were equipped with “attractive” visual analytics to focus the attention of end-users on specific parameters and events. In the *evaluation phase*, good levels of user experience versus bad level of usability suggest that end-users of *T2D Screening* perceived the potential, but they are worried about complexity. Usability and user experience were above acceptable thresholds for *T2D Care at population* and *T2D Care at individual*.

**Conclusions:**

By using a holistic approach, we have been able to understand user needs, behaviours and interactions and give new insights in the definition of effective Decision Support Systems to deal with the complexity of T2D care.

**Electronic supplementary material:**

The online version of this article (10.1186/s12911-019-0887-8) contains supplementary material, which is available to authorized users.

## Background

Most of the problems related to older age are linked to chronic diseases [1]. Healthcare systems are challenging the burden of chronic diseases by putting more emphasis on prevention, and by looking for new ways to reorient the provision of care in the light of the day-by-day collected data. A paradigm shift in healthcare delivery is required to meet these needs [2]. Individuals should be followed throughout the whole care process; their self-management role and capabilities must be clearly identified, together with the resources and services delivered by the healthcare system in relation to the stage of the disease.

Among chronic diseases, diabetes represents *one of the greatest health threats worldwide*, with 425 million people affected. The urbanization and rise of western lifestyle are accelerating the diabetes epidemic. Ninety percent of patients with diabetes have Type 2 Diabetes (T2D). The disease can be asymptomatic, slowly evolving in a first phase but then appearing with complications even before its diagnosis [3, 4].

While there is a clinical consensus on how to manage the disease through drug treatment, screening, self-management, and behavioral change, current challenges involve novel patients’ stratification strategies and effective case management, both at population and individual levels. Clinical, social, and political coordinated actions, in primary and secondary care, are required to prevent or delay T2D onset and complications. Risk prediction models could effectively contribute to support such healthcare interventions and decision-making processes [5]. However, despite the large number of models being developed and the increased interest in the clinical field, only few ends up being used in the clinical practice. Furthermore, there is a lack of consensus and examples regarding how to properly embed them into computerized Clinical Decision Support Systems (CDSS) addressing proactive search, risk stratification and case management [6–8].

In the most recent reviews about the effects of CDSS, performed in 2005 [9], 2011 [10] and 2013[11] it was shown that they are quite useful to improve healthcare processes, their impact has been constantly improving and consistent over the years, especially for diabetes management, with an increased number of interventions in primary community settings and multiple practices. The lack of solid explanations behind successes and failures has been recognized, suggesting inclusion and analysis of more dimensions, such as “system design, local context, implementation strategy, costs, adverse outcomes, user satisfaction, and impact on user workflow”.

In a more recent meta-analysis of qualitative studies, Miller et al. tried to provide contextual knowledge and possible explanations about variability of results and barriers towards deployment of CDSS [12]: they found that qualitative studies about CDSS are still scarce, and this may explain the limited understanding of actual healthcare workflows. While most studies report on alert and reminder systems, few are informing about interaction designs, naturalistic decision making, diagnostic pattern recognition, situation awareness and segments of the healthcare environments. Interaction between CDSSs and decision makers, aggregation of information to extract and present meaningful synthesis for clinical decisions and on the understanding of human reasoning and problem solving in real world settings should be further researched. The need for increased collaboration among social, behavioral, cognitive and computer scientists has been identified as a critical element to understand the main tasks, actions, and expectations of the different healthcare professionals involved in the management of chronic diseases.

Adopting human-centred design techniques can help to maximize usability, identify design goals, understand unmet needs and unsolved problems [13], and is central for work domain analysis, design and evaluation of health information system [14], as it allows understanding the cognitive work performed by practitioners in different contexts, identifying usability problems and reducing errors through task analysis, focus groups and semi-structured interviews [15–18]*.*

From a methodological perspective, the importance of combining human centred design with system engineering and design life cycles have demonstrated to increase reliability, compliance and safety of health information systems [19]. However, a great number of health information systems are not designed following human centred design guidelines, resulting in low satisfaction, acceptance and abandonment. These systems could be improved by combining different disciplines, ranging from computer to social and behavioural science [20]. Ethnographic techniques can be useful to understand that barriers for effective use of CDSS are almost non-technological [21], analysing stakeholder groups allows to understand that a CDSS may be misused or underused if their needs, knowledge and priorities are not taken into account [22], is more and more relevant in the decision making for the purchase of medical devices and for health technology assessment [23], and can have a negative or disruptive impact in practitioners routines [24].

To sum up: designing tools that support healthcare transformation for better control of chronic diseases, such as T2D, requires a holistic approach.

In this study, we leveraged on holistic, evidence-based frameworks and initiatives [25–28], to understand how risk models for T2D should be embedded in a CDSS and allow better management and coordination between healthcare providers and decision makers.

The work has been performed in the context of the MOSAIC (MOdels and Simulation techniques for discovering dIAbetes influence faCtors) project, funded by the European Commission. The consortium was composed of European institutions and enterprises combining different expertise, such as medicine, epidemiology, biomedical engineering, medical informatics, and medical technology. The goal of the project was to develop new computer models [29, 30], and implement them in tools to support the detection and prediction of T2D onset and related complications, in different healthcare settings (e.g. hospitals, clinical centres and health agencies). We integrated computer models into DSSs for T2D management, detection and prevention and studied how end-users and stakeholders interacted with the system. That is why, in the method section, we inform on how different methodologies and techniques have been selected and combined to gather perspectives from prospective stakeholders and end-users, and to define of contexts and scenarios of use. Our findings are presented in terms of user needs, implementation and evaluation aspects, and then discussed with a special attention on the cognitive and behavioural domains.

## Methods

To perform our study we leveraged on a holistic and evidence-based framework, the CeHRes Roadmap [28], built on a participatory development approach, persuasive design techniques and business modelling [31, 32] and be used by the project management team (i.e.: the team involved in the design, implementation and evaluation of the technology) as an instrument through which stakeholders can debate to clarify areas that “would otherwise remain unanswered, unclear, or unknown”, and in which “technology is not considered as a tool or end in itself, but as a catalyst for innovation” [28]. The Roadmap consists of five sequential and one iterative retrofitting phases (*Contextual Inquiry*, *Value Specification*, *Design*, *Operationalization*, *Summative Evaluation*, and *Formative Evaluation*); for each phase, a list of main research questions, tasks and methods is provided.

**Fig. 1:**
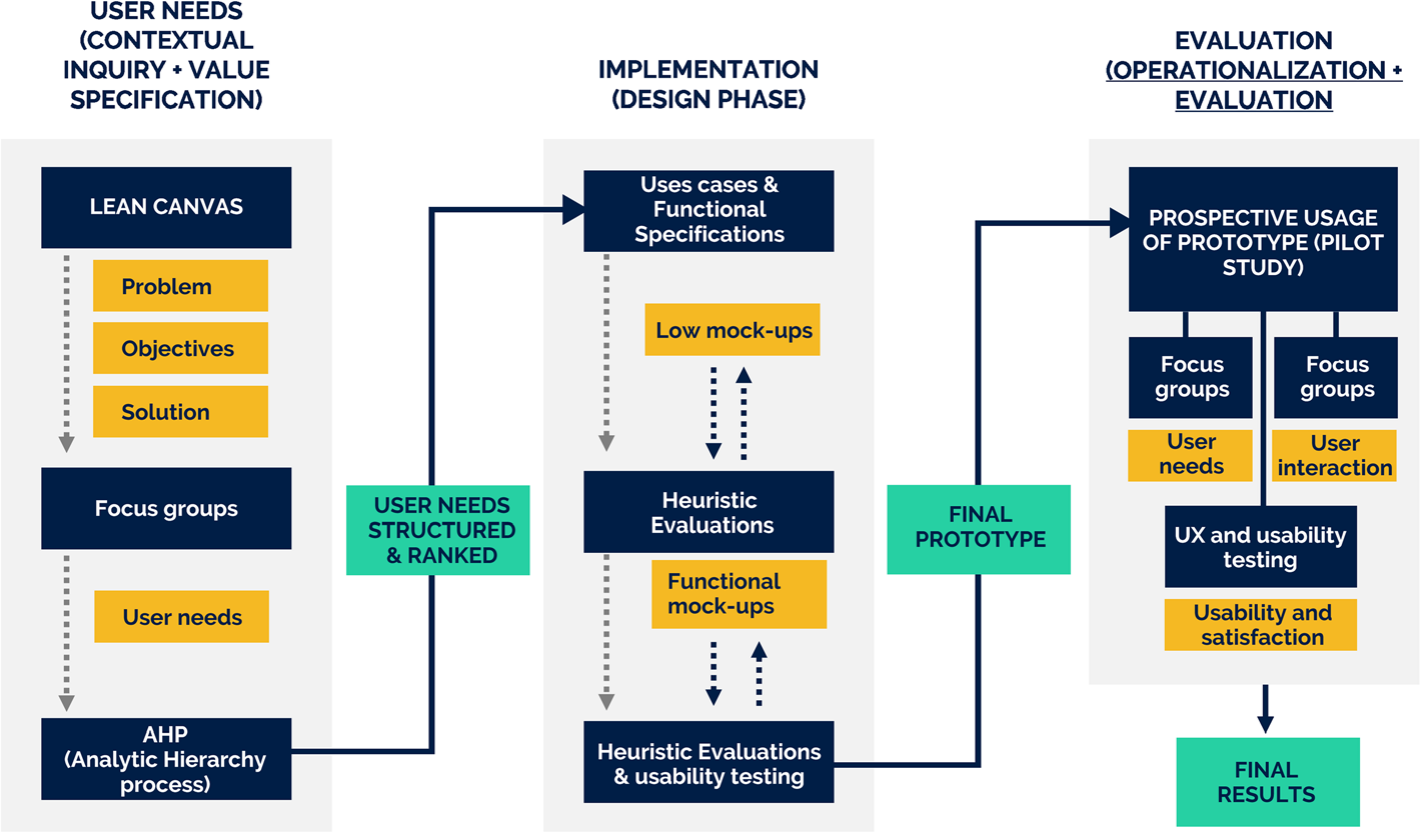
Overall view of the methodology adopted, inspired by the CEHRES roadmap and organized in three main phases, user needs (left), implementation (middle) and evaluation (right). For each phase, different methods (white rectangles) where used to get intermediate (green shapes) and main result (orange shapes)

We used the roadmap to guide the design of our methodology, organized in three main phases, as shown in Fig. 1: *User Needs, Implementation and Evaluation*. For each phase, the sequence (arrows) of use of the methods (white rectangles) is represented, together with the main intermediate (green boxes) and final (orange boxes) outputs.

### User needs

We first used the *Running Lean Canavas*, a business oriented methodology to study new ideas and build successful products [33], to understand and define from a clinical, economical and customer perspective how validated predictive models for T2D might impact current clinical practices. The most relevant output of this method is a formal definition of the problem, objectives, and solutions. Then, we carried out thematic *focus groups* to determine and describe the key stakeholders, as well as to identify and understand the healthcare improvements and to specify needs and requirements to be covered [34, 35] from the clinical, scientific and socio-economic perspective. To further study user needs and transform them into a structured set of specified requirements, the *Analytic Hierarchic Process* (AHP) was used. AHP is a multi-dimensional, multi-level, and multifactorial decision-making methodology that provides a framework for structuring a decision problem, representing and quantifying its elements, and relating those elements to the overall goals [36–39]. This method has been already used in healthcare for user needs elicitation and evaluation framework. Users of the AHP start decomposing decision problems into a hierarchy to easily include sub-problems. Two experts for each sub-problem are needed. Once the hierarchy is built, experts evaluate its elements by the means of pairwise comparisons. Once evaluations are done, experts discuss and comment the results generated, to gather insights and transform them into requirements and guidance for the development stage.

The output of this phase is a structured and balanced description of user needs, values and attributes that can be used as specifications for the implementation phase.

In our work we have used a web based online system, the AHP-OS [40]. The questionnaires have been included as Additional files 2, 3 and 4 to this manuscript.

### Implementation

Once requirements were defined, the design process started. In addition to traditional methodologies for software development, the main ideas were drafted via use cases description and functional specifications, which allowed to create and discuss mock-ups with the intended end-users. Following up on these discussions, the prototypes were refined from low-level mock-ups to functional mock-ups. The functional mock-ups were tested with Human Computer Interaction (HCI) experts first and then with end-users in close to real life situations. Both were invited in several rounds to test whether the mock-ups match usability standards, end-user’s way of thinking and working. More in detail, *Heuristic Evaluation* (HE) was used by HCI experts to identify usability issues in the existing concepts and prototypes, and to identify common usability issues before performing tests with end-users. Evaluations were performed by applying the 10 Nielsen Heuristics [41]. In turn, the concept and the first functional prototypes were refined. Then, *Usability Testing* was performed by end-users to identify improvements in the software prototypes. The usability levels were assessed through two validated questionnaires, one to measure satisfaction as perceived usability, the *System Usability Scale* (SUS) [42], the other one to assess user experience, the *Attrakdiff* [43], in line with the ISO9241.210 [44].

In the case of the SUS we have used the grading score from Sauro and Lewis [45], ranging from F (unsatisfactory) to A+ (absolutely satisfactory):

- Grade F (0–51.7)
- Grade D (51.8–62.6)
- Grade C– (62.7–64.9)
- Grade C (65.0–71.0)
- Grade C+ (71.1–72.5)
- Grade B– (72.6–74.0)
- Grade B (74.1–77.1)
- Grade B+ (77.2–78.8)
- Grade A– (78.9–80.7)
- Grade A (80.8–84.0)
- Grade A+ (84.1–100)

The output of this phase were the final prototypes, ready to be used in the evaluation phase.

### Evaluation

The main objective of this phase was to analyse the performance of the system in terms of uptake and potential impact. To assess the behaviours of end-users when interacting with the prototype, the following methods were adopted to analyse user interactions, needs and usability aspects:

- *Log files* were used to understand user interaction, collecting information about how real users were performing routine actions using the developed prototypes during their clinical practice. To facilitate the analysis of log files, an identifier for each of the actions, and for each of the situations or screens accessed while using the tool, was created. Log4J [46] and Log4Net [47] libraries were used.
- User Needs were re-assessed through *focus groups*, this time to understand the impact of the designed solution in the clinical practice.
- User satisfaction and experience were assessed through the SUS and Attrakdiff questionnaires, delivered at the end of the study. As suggested by Borsci et al., in the case of solution 2.1 and 2.2, since they were used for enough time, the learnability component has been also assessed [48, 49].

### Characteristics of participants

Table 1 summarizes the characteristics of the involved users (demographics, professional expertise and skills). Ninety healthcare professionals were involved, between four and seventeen in focus groups, and between four and nine for the heuristic evaluation, usability tests and usability questionnaires, in line with what suggested by Nielsen to spot the most important usability problems [50].

**Table 1:** Number and type of users involved in the evaluation activities

	Number and gender	Age	Years of Experience	Type of Expertise	IT Literacy (Min 0, Max 3)
Design					
Clinical Focus Group Solution 1	M = 4;F = 1	57 ± 8	30 ± 7	MD = 2, HCM = 2, N/O = 1	H = 3;M = 1;L = 1
Clinical Focus Group Solution 2	M = 5, F = 3	46 ± 14	17 ± 11	MD = 6, HCM = 1, GP = 1	H = 2;M = 3;L = 3
Clinical Focus Group Solution 1 and 2	M = 1;F = 3	50 ± 8	23 ± 12	MD = 3, HCM = 1, N/O = 0	H = 2;M = 2;L = 0
Scientific Focus Group	M = 1;F = 3	55 ± 5	22 ± 5	MD = 2, HCM = 1, N/O = 1	H = 3;M = 1;L = 0
Business Focus Group	M = 13;F = 4	46 ± 10	19 ± 10	MD = 0, HCM = 17, N/O = 1	H = 17;M = 0;L = 0
Analytic Hierarchy Process Solution 1	M = 2;F = 6	47 ± 9	20 ± 10	MD = 3, HCM = 3, N/O = 2	H = 1;M = 5;L = 0
Analytic Hierarchy Process Solution 2.1	M = 3;F = 3	54 ± 9	29 ± 10	MD = 2, HCM = 1, N/O = 3	H = 2;M = 6;L = 0
Analytic Hierarchy Process Solution 2.2	M = 2;F = 6	43 ± 12	17 ± 11	MD = 5, HCM = 3, N/O = 0	H = 0;M = 7;L = 1
Implementation					
Heuristic Evaluations (Solution 1 and 2)	M = 2,F = 3	34 ± 4	8 ± 4	BE (4), GD (1), SWD (2)	H = 5; M = 0;L = 0
Usability Test Solution 1	M = 3, F = 1	46 ± 13	14 ± 6	MD = 2, HCM = 1, N/O = 1	H = 2; M = 1; L = 1
Usability Test Solution 2	M = 1, F = 4	38 ± 10	13 ± 11	MD = 4, N/O = 1	H = 1; M = 3; L = 1
Evaluation					
Solution 1	M = 1, F = 6	42 ± 9	14 ± 10	MD = 6, HCM = 1, N/O = 0	H = 3; M = 3; L = 1
Solution 2	M = 3, F = 6	41 ± 15	13 ± 12	MD = 6, HCM = 2, N/O = 1	H = 2; M = 5; L = 2

*M* Male, *F* Female, *BE* Biomedical engineer, *GD* Graphic designer, *SWD* SoftWare developer, *MD* Medical doctor, *HCM* Health care manager, *GP* General practitioner, *N/O* Nurses or others; *H* High, *M* Medium, *L* Low

Clinical focus group and Analytic Hierarchy Process involved between six to eight individuals (at least two per each domain). They were medical doctors, healthcare managers, nurses or other type of professionals involved in T2D care (e.g. case managers, nutritionists). Software developers, biomedical engineers, graphic designers experienced in the adoption of human computer interaction methodologies, were involved in the heuristic evaluations. In the business focus group, most of them were healthcare managers. In none of these evaluations there was the need to have an informed consent or approval from ethical committee. While in focus groups the average age is around 50 years with more than 20 years of experience, in the evaluation the medium age is 42 years with about 14 years of experience. The IT literacy is medium or high for almost all the users, with some exception with the clinical focus group for solution 2 and for the users involved in the evaluation phase.

## Results

Results are presented for each one of the three phases.

### User needs

#### Lean canvas

Domain experts were involved in approaching the problem from different perspectives (e.g.: technological, clinical, epidemiological, industrial, etc.) until a consensus was reached. They recognized that current diagnostic criteria are biased on microvascular complications, missing to identify early symptoms of the disease. Two types of DSS were proposed:

- a DSS for T2D Screening, hereinafter also referred as *Solution 1*, for detection and prediction of the onset of the disease: a system to be built on top of intelligent risk-scores based on probabilistic models derived from dataset that, at present, are almost available from clinical studies;
- a DSS for diagnosed T2D patients, hereinafter also referred as *Solution 2*, making use of longitudinal healthcare records to derive trends and patterns through data mining techniques for better characterization and prediction of complications: two subsets of solutions were further identified, one, *CDSS for T2D Management* (hereinafter also referred as *Solution 2.1*), to be used at population level among healthcare managers and their teams, the other, *CDSS for Follow-up visits* (hereinafter also referred as Solution 2.2)*,* by practitioners during individual visits. Solution 2 is described in [51].

#### Focus groups

The vision, objectives and high-level solutions were discussed with end-users and stakeholders, to derive user needs, values and expectations. Two clinical focus groups with healthcare practitioners from the Madrid regional healthcare service, Spain, and from the private and public healthcare system of the Pavia province, Italy, were carried out to discuss Solution 1 and Solution 2 respectively. A third focus group was carried out with healthcare professionals from the Valencia regional healthcare service, Spain, to confirm the results of the previous ones. Additionally, two scientific focus groups were held, as well as a business focus group oriented to explore the market potential. The full results are detailed in the Additional file 1. Regarding Solution 1, experts agreed about the importance to have tools that allow to exclude those persons that are not at risk of developing the disease (i.e. false positives) and therefore reduce the unnecessary activities. The CDSS should be linked with existing registries and allow definition of lifestyle interventions to mitigate the detected risks. Several participants expressed concerns about the increased workload and additional costs caused by such tools. Regarding Solution 2, pattern recognition and trends should be used to give an overview about what is happening in a health unit, to improve the efficiency of the provided services and to identify gaps between clinical guidelines and real practice. From a scientific perspective, reluctance to use information from subjective assessments to build the models (e.g. patient diaries) and about fixed time for predictions was clearly expressed. Using medications patterns and Continuous Glucose Monitoring as proxy for complications was considered as innovative and promising approach.

#### Analytic hierarchy process

**Fig. 2:**
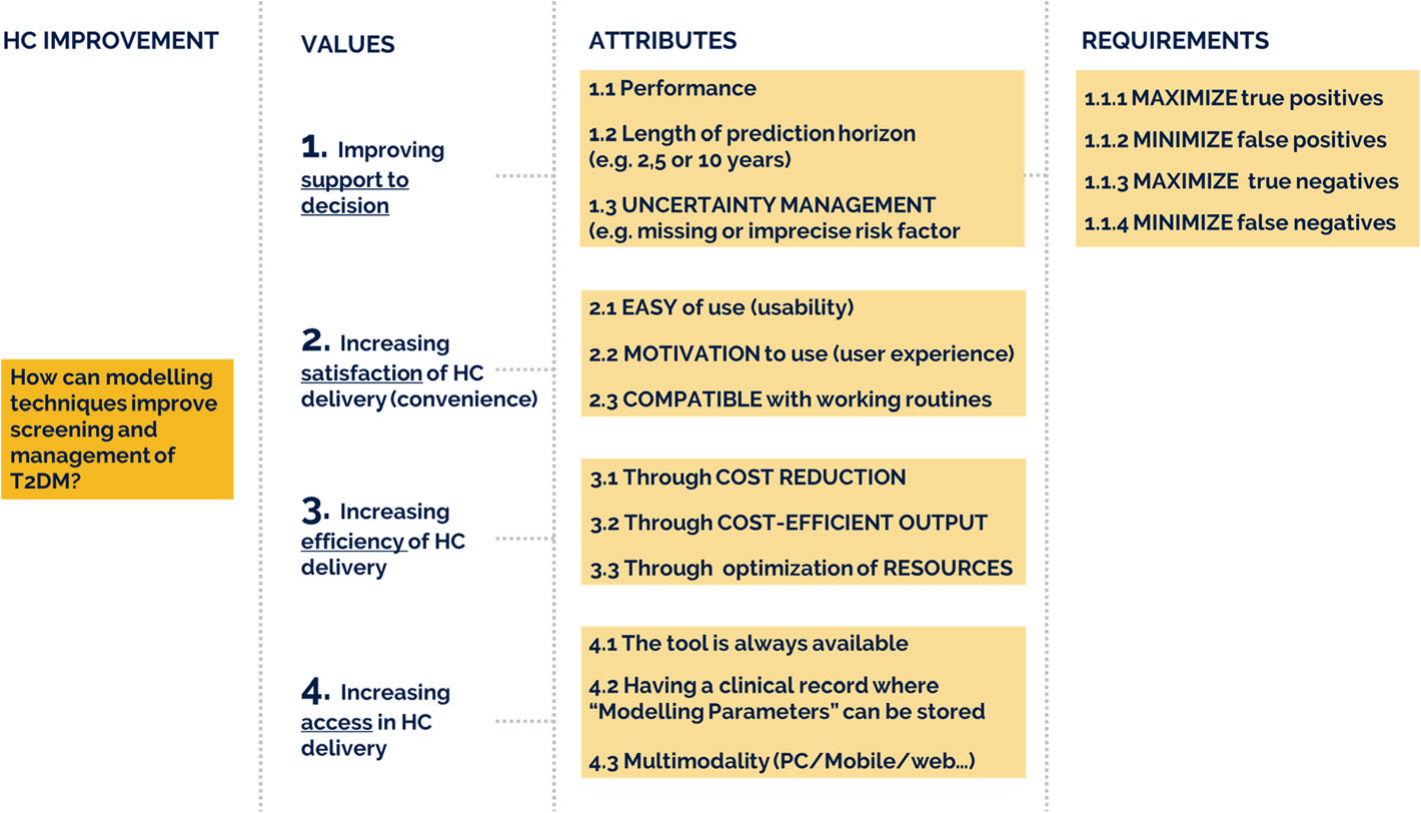
Hierarchy of needs for the development of the MOSAIC Tools

**Fig. 3:**
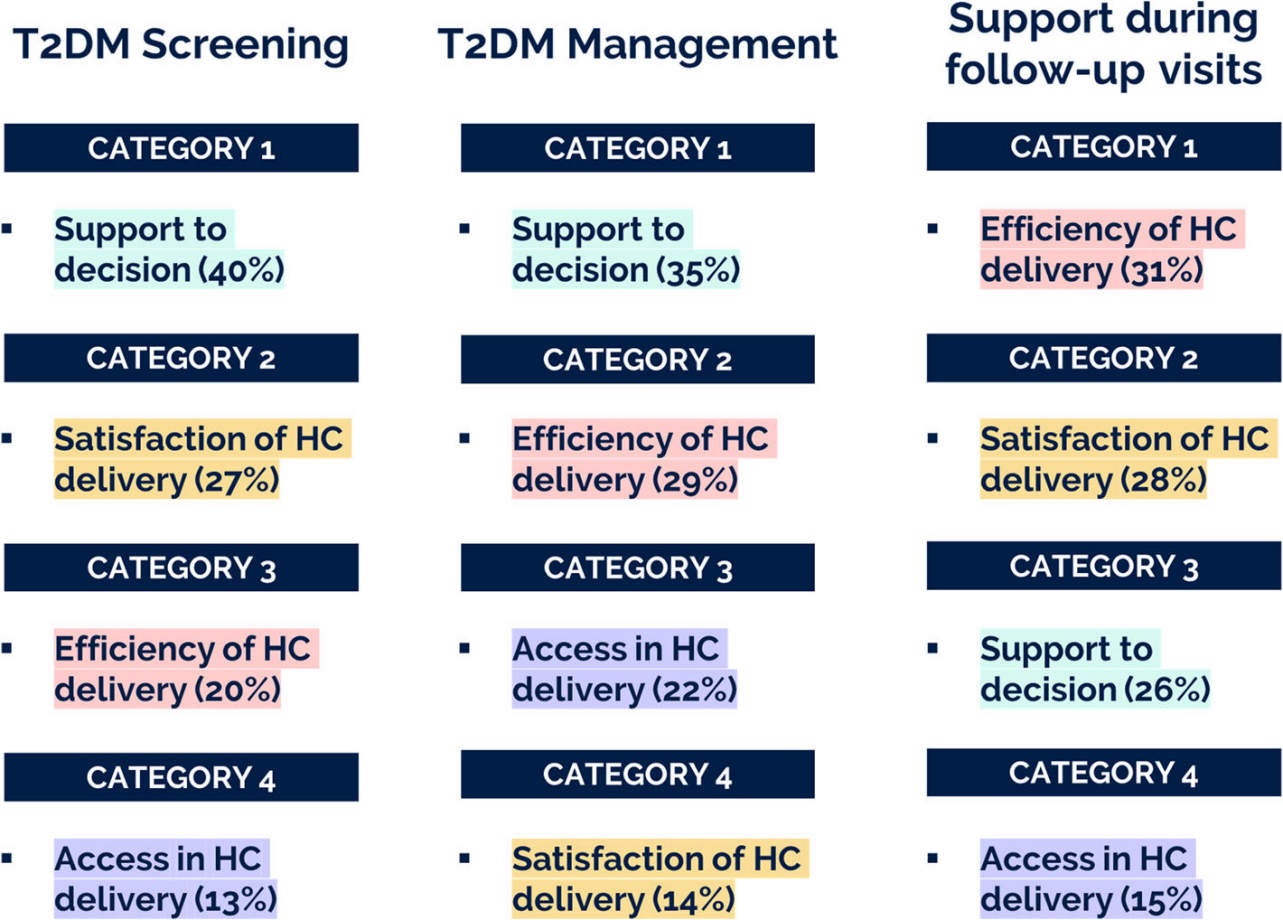
First Level of Needs evaluated by end-users of *Solution 1 – TD2M Screening* (*n* = 8), *Solution 2.1 – T2D Management* (*n* = 6) and *Solution 2.2 - Support during Follow-up visits* (*n* = 6). It can be observed how the same needs (highlighted with the same color) have been ranked differently for each solution

**Fig. 4:**
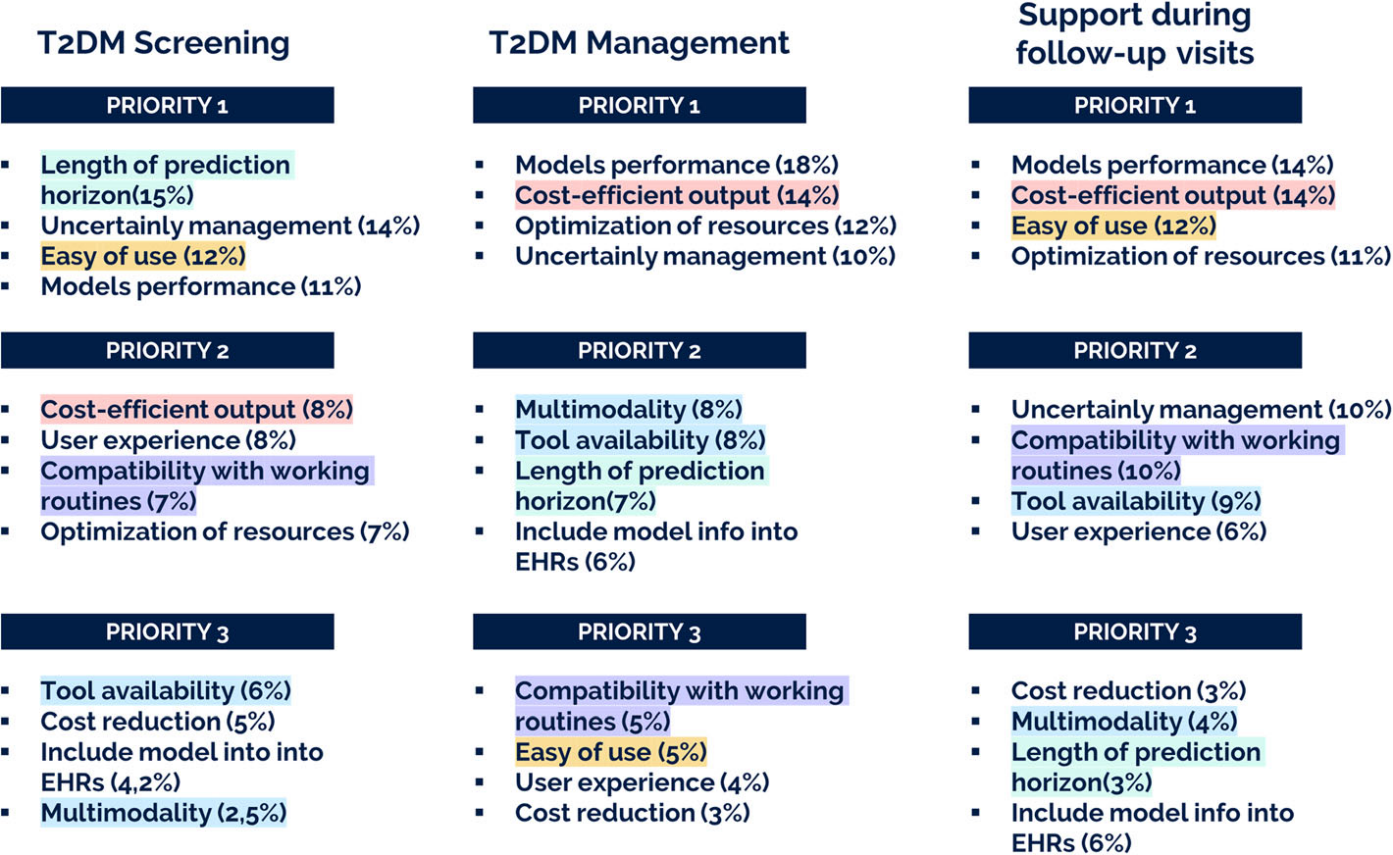
User Need priorities for Solution 1 - T2D Screening, Solution 2.1 - T2D management and Solution 2.2 - *Support during Follow-up visits*

The creation of the hierarchy, already described in [52], helped to redefine, categorize and structure the outputs of the previous activities into a hierarchy of needs, shown in Fig. 2, and ranked by experts for solution 1, 2.1 and 2.2, as a reference for discussions and comparison of the priorities and future directions to be taken for the implementation, evaluation and exploitation of the tools. Four main categories of needs, *Improving Support to Decision, Increasing Satisfaction, Efficiency and Access of Healthcare delivery,* were identified. Three groups of end-users ranked needs for each solution, as shown in Figs. 3 and 4, illustrating how the 1st level (categories of needs) and 2nd level (needs) were rated.

As *T2D screening* and *T2D management* are largely focused on taking decisions related to risk-stratification strategies and allocation of resources, one of the most important need is the *Support to Decision*, while *Efficiency of Healthcare (HC) delivery* is the most important need to assist diabetes specialists or general practitioners (GPs) during follow-up visits. The second most important category for *Support during follow-up visits* is related with the impact on the daily routines of professionals, *Satisfaction of Healthcare (HC) Delivery*, while this is not the case for *T2D Management*, considered as the least important category. *Efficiency of Healthcare (HC) Delivery* is the third category for T2D*Screening*, while it is the second for *T2D Management*. *Access in Healthcare (HC) Delivery* is the least important category for *T2D Management* and *Support during follow-up visits*, while it is more important (third category), in the case of *T2D Management*.

About Solution 1, we can appreciate from Fig. 4 that *length of prediction horizon*, *uncertainty management*, *usability* and *model performances* are the most important needs. For Solution 2.1, *model performances* and *uncertainty managements* are important, however *cost-efficient output* and *optimization of resources* are the top ones. In the case of Solution 2.2 there is a mix of needs like Solution 1 and Solution 2.1. *Usability* is highly important for Solution 1 and Solution 2.2, while has a lower level of importance for Solution 2.1. *Tools Availability*, *multimodality*, and *compatibility with EHRs* are ranked as less important for Solution 1 and Solution 2.2, and as medium important for Solution 2.2.

### Implementation

Use cases (UCs) and functional specifications were developed together with the definition of low-level and functional versions of the mock-ups.

In the case of *T2D Screening*, three main UCs were defined: *UC1.1-Risk Factors and Indicators to be adapted to Public Health*, to be used by local, national and regional healthcare agencies, which usually have demographic information available to perform population screening. *UC1.2-Risk Factors and Indicators to be adapted into ambulatory settings*, to be used at individual level in primary care by GPs. *UC1.3*-*Risk Factors and Indicators to be adapted to citizens for personal use*, in this case to be used as a self-screening tool promoted by healthcare systems, professional societies, or patient’s organizations.

In the case of T2D care, the UC resembles what identified in the need phase: *U2.1-Top-down analysis for decision makers*, to be used by managers in primary care or secondary care units as a drill down, business intelligence tool at a population level. *UC2.2-Decision support for clinicians*, in this case, trends and patterns should be mined at individual level, using information available in a primary or secondary care unit and support practitioners. More details on the UCs are provided in the Additional file 1.

All the UCs were developed and tested with human computer interaction experts and end-users. In the case of UC1.3, it was decided to wait for final and full validation of the predictive models before starting to implement it, as it directly involves patients, and it is not included in this work.

During the implementation process, two iterative rounds of development took place, resulting in a low-level mock-up and functional mock-up respectively. The first mock-up was evaluated through heuristic evaluation, while the second was also evaluated through usability tests with real users (their profile is provided in Table 1).

The most relevant usability aspects emerged during the first evaluation were related with the most common usability errors (*H1 - Visibility of system status, H2 - Match between system and the real world* and *H7 - Flexibility and efficiency of use*). Experts identified the importance of decomposing the process that the tool is supporting into smaller steps, while giving them a clear picture about the whole process, and where she/he is at a given moment (e.g. by using breadcrumbs). They also suggested to inform users on the expected event upon clicking a certain button or link through clear text labels, as icons may be not enough self-explanatory.

During the second heuristic evaluation, common mistakes were reduced, while usability errors related to the healthcare process increased (*H2 - Match between system and the real world* and *H3 - User control and freedom* were the most frequent in this case). The most important finding is that users want assisted guidance about the steps needed to perform screening or risk-stratifications and for this reason they need clear description of tabs, filters and generated outputs. More details about this study have been described in [51–53].

The needs identified during this phase were shared by all the cases and include: the need to increase personalized filtering and stratification functionalities, to provide visualization options, to automatically build and send reports, to include additional contextual information and explanations, and to provide more aggregated and less complex information.

**Fig. 5:**
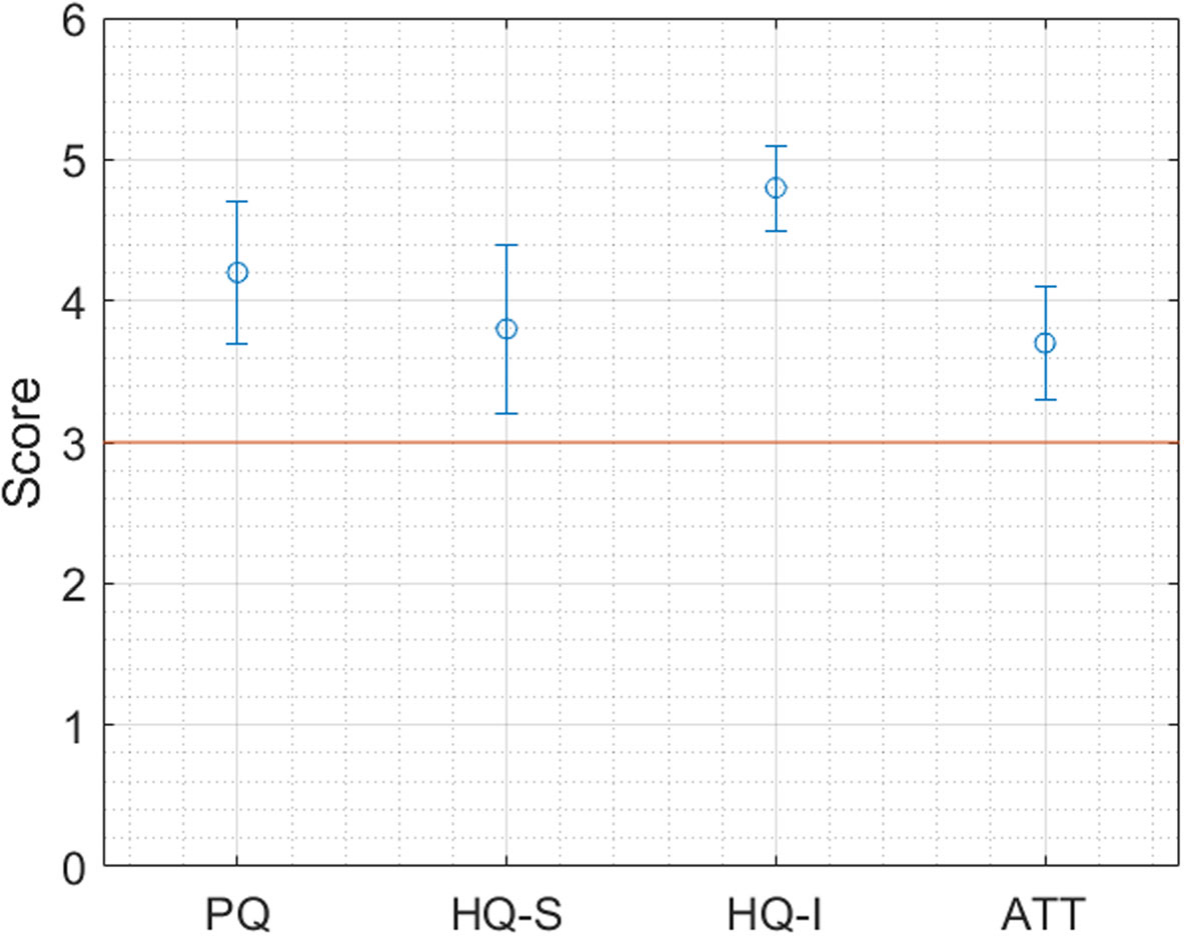
User Satisfaction levels (means and Confidence Interval) for Solution 1, assessed through the Attrakdiff questionnaire

**Fig. 6:**
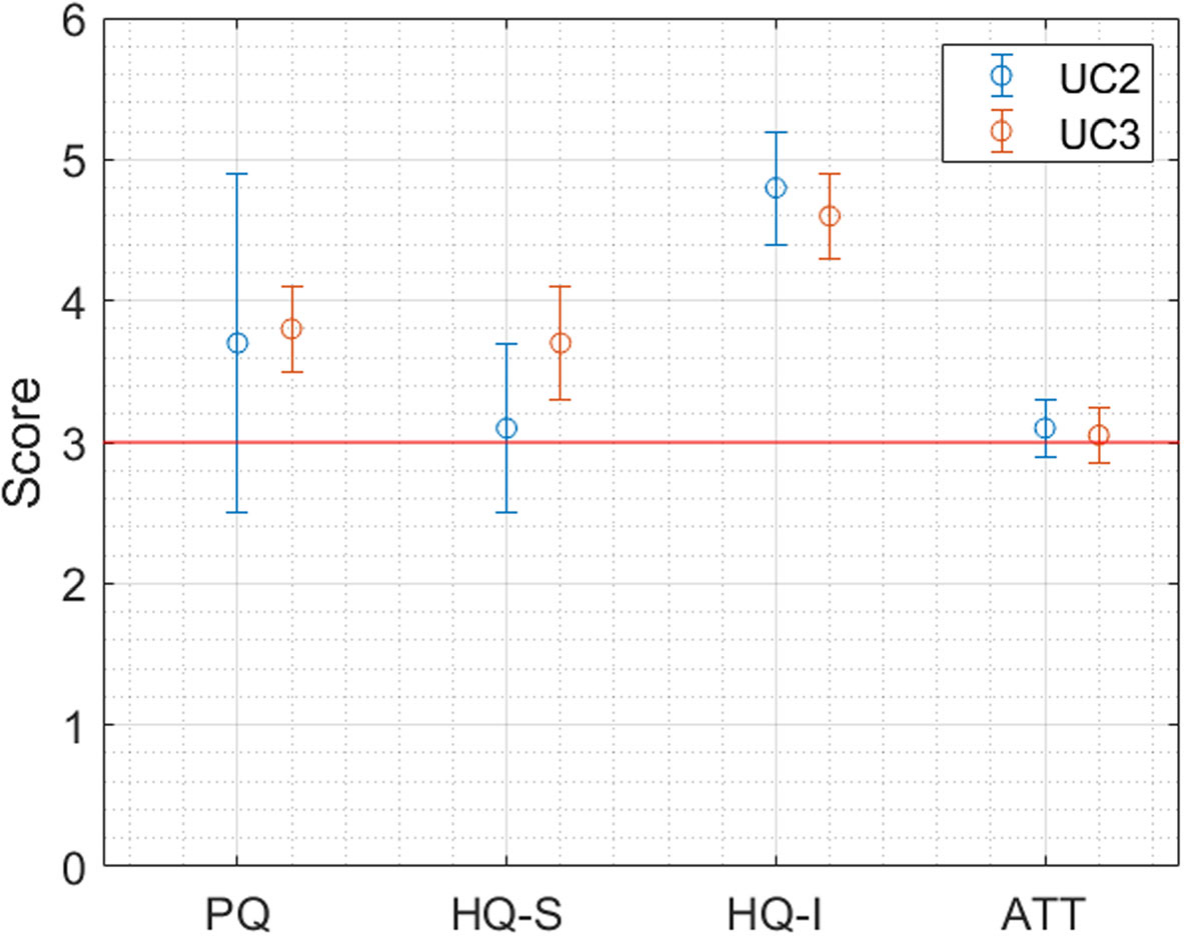
User Satisfaction level for Solution 2.1 (UC2) and 2.2 (UC3), assessed through the Attrakdiff questionnaire

Figures 5 and 6 summarize the results of the Attrakdiff for Solution 1 and 2: our quality criterion consisted in having the Confidence Interval of the mean value not touching the scale’s middle score of 3. The results for Solution 1 are above the minimum threshold, meaning that they are “not frustrating” end-users. The overall SUS Score is about 61, corresponding to the D grade. The main issues that were pointed out regarded the need of a technical support to be able to use the system, the lack of confidence in using it, and the necessity to learn too many things before being able to use the system. Considering that a score above 80.3 is needed in order to have highly usable products, the usability of the MOSAIC tools for Solution 1 has been perceived as low by primary care and healthcare agency users.

As regards Solution 2.1, as shown in Fig. 6, only the identity hedonic quality is statistically above 3. In the case of Solution 2.2, the pragmatic quality, the stimulation and identity hedonic qualities are statistically above 3, while the attractiveness is around the value of 3.

As regards the perceived usability, the SUS score was 81.5 ± 7.62 (86.25 ± 8.83 for 2.1 and 78.33 ± 6.29 for 2.2).

The most important problem in the case of Solution 2 were inconsistencies found while using the system. The results of these tests lead to several and important changes on the prototypes. Within Solution 1, filtering options were added, while interactions and displayed information were made clearer. A new functionality to create reports for the users of the different Use Cases was added.

In the case of Solution 2 interactions with the users were simplified by means of aggregated visualization features: traffic lights were added, to capture the attention of the care provider in case a negative trend exists (e.g.: increased glycated haemoglobin (HbA1c) level, decreased adherence to drug, etc.).

### Evaluation

Once the final prototype was ready, the solutions were tested in two different healthcare settings. Solution 1 was tested in the endocrinology department of a public and reference centre for T2D, Hospital La Fé, in Valencia, Spain. Solution 2 was evaluated during a pre-post study in the Endocrinology department of the Istituti Clinico Scientifici Maugeri Hospital of Pavia (ICSM), Italy. The clinical impact is not reported in this work: as we focus on qualitative aspects, describing the most relevant information about user reactions, behaviours and needs. Results on clinical impact have been reported in [51]. In both hospitals, the studies were discussed and approved by the Institutional review boards.

### Solution 1 (T2D screening tools)

To perform a realistic evaluation of T2D screening tools, we defined two Validation Cases, one for healthcare agency settings (VC1) and another, organized in three sub-scenarios, for clinical settings (VC2).

- VC1 – T2D Prediction Tool for Health Care Managers, to predict how many patients are at risk of developing T2D and eventually design intervention strategies at regional level.
- VC2.S1 – T2D Prediction Tool for Clinicians, to predict how many patients in a healthcare unit are at risk of developing T2D and design interventions at unit level.
- VC2.S2 – Case Titration, to validate the system response. This tool is used by a doctor or by a nurse, who has a list of patients at risk and its goal is to help users to validate the risk of T2D suggested by the system.
- VC2.S3 – T2D Detection and Parameter Estimation Tool, estimating the risk to develop T2D within the next 12 years for each patient, based on the automatic estimation performed on the values of clinical and demographic variables that have no value and based on the estimation of oral glucose tolerance test and high density lipoprotein cholesterol.

#### Usage of the tool through log file analysis

The tools have been used for 2 weeks in May 2016. Eleven professionals used the tools (Table 1); statistics about number and duration of sessions as extracted from the system log files are shown in Table 2: the tools were used between two and three professional per day, for about half an hour, with six patients evaluated per user, ten patients per day.

**Table 2:** Distribution of sessions (number, duration, number of patients per day and per session)

	Mean	SD	Min	Max
Number of users per day	2.5	1.6432	1	4
Duration of sessions (min)	26.16	13.7150	0.25 (≈15 seg)	45.93
Number of patients evaluated per user	6.25	4.9749	1	15
Number of patients evaluated per day	10.7143	12.1890	0	26
Number of sessions per doctor (user)	1.8182	1.1677	1	5

The most accessed case was the one related to T2D Detection and Parameter Estimation Tool (45% of the times), followed by Case Titration (34%), T2D Prediction Tool for Clinicians (16%) and T2D Prediction Tool for Managers (5%).

#### Overall feedback from focus groups

Solution 1 represents a change in terms of organizational and procedural aspects as it triggers an active risk stratification of T2D (in primary care, secondary care or healthcare agencies). The action required to introduce an effective risk stratification tools for T2D regards an active collaboration with the healthcare organizations available to implement this innovation. This involves generating data warehouses specific for this purpose (upon a process of data pooling and quality checks) and the training of healthcare professionals. The personnel involved in the evaluation has provided feedback on how to improve some specific aspects, such as: body mass index (BMI) categories should meet the WHO recommendations; the decision-making process for risk predictions should be based on the comparisons with what detected by professionals; recommendations should be displayed in checkboxes, so to better select them depending on the risk estimation and the imputed variables; more filtering options should be included (e.g. erectile dysfunction).

#### Usability and user experience via questionnaires

**Fig. 7:**
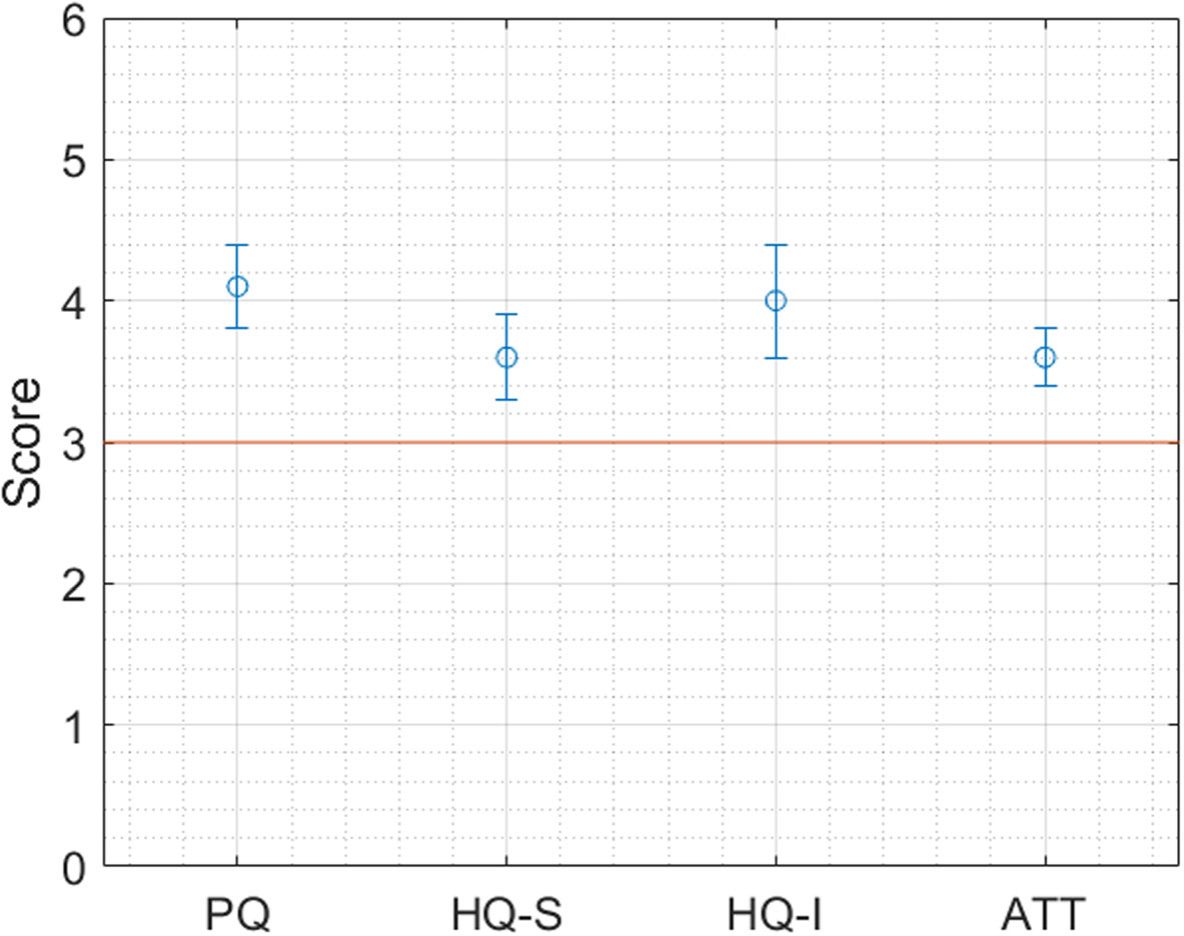
User Satisfaction levels (minimum acceptable threshold is 3)

User satisfaction levels, evaluated through the Attrakdiff questionnaires, were found above the minimum threshold of 3, meaning “almost very good” for all the 4 subordinate constructs (*pragmatic quality*, *stimulation* and *identification*, and *attractiveness*), as shown in Fig. 7.

Perceived usability was 55.36: this value according to Sauro&Lewis can be interpreted as a grade of a D. The main issues pointed out in this case regarded the need of a technical person to be able to use the system, the perception of the system as cumbersome and the necessity to learn too many things before using the system.

We can conclude that MOSAIC T2D tools for Solution 1 have good levels of user experience but low levels of perceived usability; they are introducing a breaking and disruptive routine and therefore more work and efforts in terms of user training, and set up of organizational and procedural measures.

### Solution 2 (evaluation of T2D management tools)

The tools were evaluated at Istituti Clinico Scientifici Maugeri Hospital of Pavia (ICSM), Italy, for Solution 2.1 (population management) and 2.2 (clinical decision support for single patients during visits). Solution 2.1 and 2.2 have different settings, employments and users. The implemented evaluation strategies, while following the general proposed schema, required specific adaptations. In the Solution 2.1 the focus was to evaluate the introduction of a new process that was not possible earlier for diabetes patient management. In the Solution 2.2, the evaluation of the impact was performed both during clinical activities while monitoring clinicians using the tool and by performing usability testing. In the following, we illustrate the results of the evaluation activities using the log files of the system, focus groups and questionnaires.

#### Usage of the tool through log file analysis

The prototype was tested in a real clinical and health care environment for a period of 55 days (January – April 2016). Once having been trained about the system functionalities, clinicians were asked to use the tool during their daily activities, for the management of the ward and during follow-up visits. A total of 305 sessions were recorded (being a session defined as the continuous time interval that a user has used the tool, considering as the *start* event the access to the tool, and as the *end* event the last interaction with the tool, defined as the latest interaction before a non-continuous use of the application took place). The results show that Solution 2.2 (patient management during visits) were more extensively used than solution 2.1 (population managements). The 55% of the accesses to the system were related to Solution 2.2. Table 3 shows statistics regarding the number of sessions per day, their duration, and number of patients visited during follow up visits at the hospital, assessed through the evaluation of Solution 2.2.

**Table 3:** Distribution of sessions (number, duration, number of patients per day and per session)

	Mean	SD	Min	Max
Number of sessions per day	5.3509	2.7870	1	14
Duration of sessions (min)	47.4314	88.9975	0 (22 s)	465.8000 (≈ 8 h)
Number of patients per session	1.5574	1.3780	0	9
Number of patients per day	8.3333	4.5878	1	28

We collected and studied the number of times users accessed the main menu options and the main modules, and for each module the number of times they accessed specific functionalities. Specifically, the implemented final prototype of Solution 2.2 is structured in three main screens representing risk factors and their evolution in time, clinical variables time series and drug purchase patterns. The access distribution was uniformly distributed among three screens: 30% for the risk factor screen, 32% for the drug exposure, and 38% for the clinical variables screen.

#### Overall feedback from focus groups

We performed two evaluation sessions involving three clinicians and one healthcare policy maker. During these sessions, clinicians were asked to use solution 2.1 and compare snapshots of the MOSAIC population across different time periods. We considered the population at the beginning and at the end of the validation study (January 2016 and April 2016). The main *clinical comments* on the center population regarded:

- The usefulness of the tool to inspect specific clinical questions. Participants highlighted the importance to identify sub cohorts of patients who experienced a specific acute event to inspect possible noncompliance to guidelines.
- The possibility to analyze disease progression through longitudinal data representation as a function of hospitalizations and disease uptake. They were able to cluster specific groups of subjects while assessing disease complexity, they found this functionality crucial to understand the treated population.
- The comparison of the center population at two different time points. A comparison of the population between the start and the end of the validation period highlighted a significant increase in the number of patients with high cardiovascular risk. This result stimulated clinicians to in-depth inspect how the cardiovascular risk evolved in time, to understand which groups of patients reached the maximum risk level, and to formulate hypotheses on the detected situation.

Several useful comments were also made regarding the *functionalities* of the system. Users were interested to (i) refine tuning features for customized filtering and temporal analyses functionalities, (ii) personalize the display of the results and (iii) add further information and statistics to answer specific research questions on the population.

#### Usability and user experience via questionnaires

**Fig. 8:**
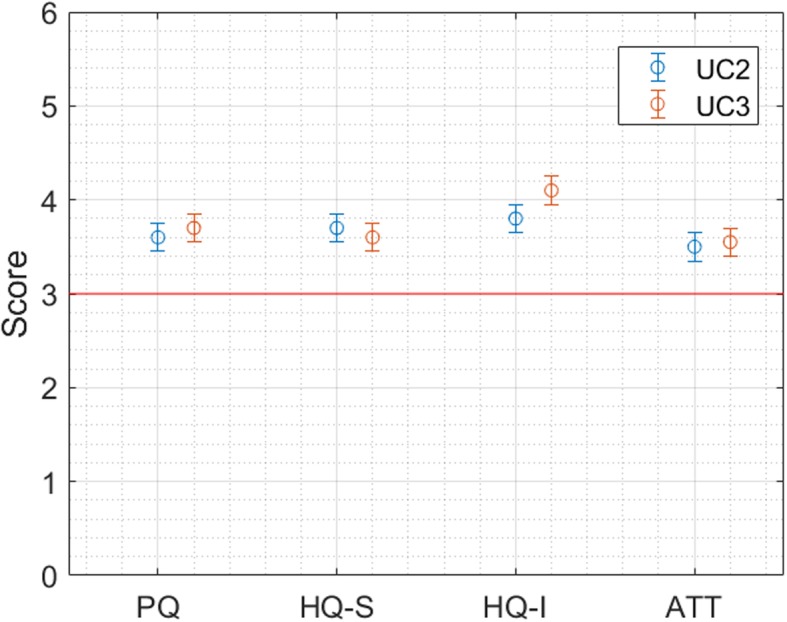
Results of the AttrakDiff Questionnaire for Solution 2.1 (UC2) and 2.2 (UC3)

In both solutions the user experience is “very good”, as it is above 3 and almost above 3.5 for all the dimensions, about 0.5 better than the previous evaluations, as shown in Fig. 8.

Similar results are observed also for the SUS scale. The overall SUS Score is 79.32, corresponding to the grade of B. System learnability was scored as 3 (STD 1.2 for solution 2.1 and 0,8 for 2.2).

To conclude, the usability and learnability scores for Solution 2 are quite good. However, it was not possible to make a 3rd round of developments, following the usability recommendations provided after the usability tests and this could explain the slightly reduced score from the tests (where an 81.5 score was obtained) to the final evaluations (79.3).

## Discussion

In this work we have presented a holistic approach to design, implement and evaluate IT solutions to tackle the epidemics of T2D. This approach, applied in the context of the MOSAIC project, was based on the CeHRes Roadmap, which has been further elaborated to precisely define the sequence and combination of actions and methods to be executed at each step.

This mixed method approach allowed to acquire all the necessary information during a short period of time, thus optimally supporting the development process. Similarly to relevant literature in cognitive informatics [13], we have focused on diverse stakeholders to catch different perspectives, relied on multiple methods to make them more reliable and discovered elements, properties and perspectives which are more difficult to extract with traditional methods: If, from the functional and technical side, the requirements can be gathered through traditional software engineering techniques (like Use Cases development), other techniques coming from human and social sciences were adopted to meet the needs of end-users. It is important to perform usability tests before the final validation, in order not to compromise its success. If the prototype turned out to be not usable enough for many users, the whole evaluation would be jeopardized. The process that led to the development and implementation of the system prototype involved end-users and stakeholders in the whole analysis through multiple and small tests as recommended by Nielsen [50].

Given the multidisciplinary nature of T2D care and its challenges, and the need to find a balance on how much effort can be dedicated to the reiteration of the development phases, the holistic and flexible nature of the CEHRES roadmap was useful. Holistic means that system designers, clinicians, biomedical engineers, but also health economists and business developers, should actuate synergic strategies to develop integrated solutions and avoid isolated activities. Since a holistic approach can be counterproductive if not accompanied by a redundant strategy, multiple and repeated tests are needed before drawing a consolidated “holistic” conclusion, whether it is on user needs, implementation or evaluation aspects. We think that our approach is quite complete and comprehensive: from a human centered design perspective, it allowed identifying topics and collecting insights to better understand the barriers towards CDSS implementations, letting users to achieve their goals, as identified by [15–18].

To our knowledge this is one of the first works focused on understanding user needs, behaviours and expectations and corresponding requirements for CDSS built on top of predictive models and data mining techniques for chronic diseases. For this reason, we have reported results in the three main phases, User Needs, Implementation and Evaluation.

Regarding the *User Needs phase*, we have highlighted the importance of conducting multiple focus groups to catch different perspectives before drawing a list of consolidated user needs and values. We have used Lean Canvas to formalize the problem, objectives and solutions, and to identify customer segments. User needs were put in hierarchical order thanks to the AHP. Similarly to [54] the identification of value at early stage with has been considered as vital to drive the design of our solutions and have evidence-informed value proposition. However, in our case, the literature review was replaced by a “Scientific focus group”, where experts helped us to align our innovations to current practice and guidelines.

We have found that prediction models for the onset of T2D can be introduced in the clinical practice by understanding the data that they can work with: these models can be built, trained, and validated on data coming from clinical studies, as the current healthcare systems are not (still) producing reliable and longitudinal information on people at risk of T2D. On the other hand, information on people already diagnosed with T2D does exist and it is produced by healthcare information systems, but mainly for administrative and financial purposes: the challenge is how to pool and aggregate information that can be relevant for clinical practitioners. These findings allowed us to focus on the definition of operative solutions and objectives around these two main activities: Solution 1, using predictive models built on top of existing clinical studies on T2D onset. Solution 2, using data mining techniques on top of existing longitudinal electronic health records data. The execution of multiple focus groups helped us to gather and consolidate user needs for each solution. In some cases, the information collected was even contradictory: for instance, in Solution 1, some end-users wanted the system to focus exclusively on identifying False Positives, others on True Negatives, others were even scared about a tool that could support in finding new cases of T2D. This highlights the perception that the discovery and management of new cases for T2D could introduce a novel routine (that was referred to as *active search*) in the existing healthcare system.

Within Solution 2, we faced a different situation: while guidelines and recommended actions on how to manage T2D are available, the challenge is to deliver reliable and meaningful information to daily practitioners and managers. During the first phase, we realized that the offer related to Solution 2 should be split into two sub-solutions, the first giving support at a population level (Solution 2.1) and the second at an individual level (Solution 2.2). Once the problem was de-structured and analysed as different sub-problems, we used AHP to recompose the puzzle and have a consolidated view of user needs, objectives and problem. We ranked priorities for all the solutions, and we found that in some cases solution 1 and solution 2.1 had similar priorities, since they both deal with support on risk-stratification decisions. Solution 1 and solution 2.2 have in common the importance of not introducing a negative impact on the daily activities of health care professionals. The user satisfaction dimension is extremely important: in the first case because we are introducing an “extra” activity (*active search* of new T2D patients) within the clinical workflow, in the second because we should reduce the burden of GPs and specialists in following up a multitude of T2D patients. Furthermore, we noticed that HC managers give more importance to have tools available every time and everywhere, while professionals involved in T2D management and screening do prefer an easy to use solution.

All the previous findings were used to develop our solutions during the *implementation phase*. We defined 3 UCs for Solution 1, being performed at multiple levels (e.g.: primary and secondary care, healthcare agencies, citizens, etc.), while Solutions 2.1 and 2.2 have been mapped to 2 UCs, focused on primary and secondary care settings. Lean Canvas helped us to formalize use cases, beneficiaries and end-users. Two rounds of HE allowed us to reduce common usability errors and focus on improving usability aspects related to the healthcare process we are introducing with our tools. Even with a well-defined development plan, IT experts can unconsciously underestimate the importance of providing clear and continuous guidance to the end-user of the tool during each moment of the interaction. The improvement of these aspects prior to the conduction of usability tests allowed us to find further improvements that were more related with their real needs. In the case of solution 1, we could clearly identify and design specific moments that are relevant to the active search for T2D: gathering structured input information, running ad hoc risk models, interpreting their results and take actions according to the predicted scenarios. We could understand that the configuration of the tools can be different for each healthcare setting. For example, a public health agency has less information that is usually provided at population level. Models in this case should work also when information is not continuously available, and the most relevant action is the connection with primary and secondary care centres that can continue the active search in their settings. In the case of solution 2, even though we could provide users with suitable filtering options and valuable information, they were still not convinced about the effective introduction of our tool. For this reason, for solution 2.2, we introduced attractive visual analytics (using the *traffic lights* representation) to catch the attention of end-users and make them focus on specific parameters. For solution 2.1, we understood that we needed more efforts to further simplify, aggregate, and synchronize the information coming from different sources (e.g. drug intake patterns, appearance of complications, diet levels, etc.), and we focused on providing end-users with a way to dynamically formulate clinical questions through the tool.

About *the evaluation phase*, the first finding is about the difference of studies that were chosen to evaluate the two solutions. Given the innovative aspects associated to Solution 1, it is difficult to find centres that have available and reliable information to screen for T2D, as well as human and financial resources to perform active searching. For this reason, the evaluation study had an exploratory nature, to understand the reactions of end-users to this kind of innovative software solution. We thus introduced *Validation Cases* to simulate actions and interactions between users and units. In this phase, the most important feedback related with T2D Screening is that we were introducing a new process, rather than facilitating or supporting the existing practice. This means that users need to learn and understand how this tool can be related with their daily activities. The resulting good levels of user experience versus the not good level of usability suggest that they perceived the good potentials of the tool, but they are worried about the complexity it adds to their working routines.

In the case of Solution 2, one of the most interesting results is related to the interest of end-users in the possibility of using Solution 2.1 functionalities to answer specific clinical questions, related either to specific conditions (e.g. myocardial infarction, poly-medicated patients) or to the evolution of the disease in terms of complexity, which might be relevant to evaluate the specific features of the care center. Starting from a selected population, it is possible drilling-down to more detailed information up to individual cases, using Solution 2.2.

Even if end-users expressed the need of more personalization and filtering options, in both cases the usability and the user experience are all above the acceptable thresholds.

The proposed holistic approach is aimed at helping IT innovators in providing solutions for the multiple end-users, stakeholders and healthcare units that should be involved in the continuum of care, early detection and risk stratification of prevalent chronic diseases such as T2D.

Our study has several limitations: first, we were not able to prospectively evaluate the final prototype of solution 1 in primary care settings and healthcare agencies; to minimize this limitation we involved these types of users in single sessions of usability tests. Second, we were not able to finalize the developments of the final prototype for Solution 2; this would have allowed us to have even better usability score. These limitations, which are typical of interdisciplinary and international research projects, recall real life situations, when the project management team must take decisions that may positively affect some aspects and negatively influence others. It is important to take consensual decisions and give preference to multiple, multifaceted, stepwise short but focused evaluations, rather than wide but dispersed ones.

## Conclusions

In this study, a holistic approach was adopted and contextualized to design a system able to turn computerized modelling techniques into IT tools that support decision makers in T2D. Starting from the CeHRes roadmap, we have defined a methodology that details the sequence and composition of multidisciplinary methods (Lean Canvas, Multiple Focus Groups, Heuristic Evaluation, Usability Testing, User Interaction analysis) and is organized in three phases (user needs, implementation and evaluation). These phases are all equally important to gather information and to understand end-user needs, their behaviours, reactions and expectations with the technological solution that is iteratively provided to them. In future works we will focus on the results of the evaluation, highlighting the clinical impact of the MOSAIC solutions. Our approach was useful to clarify which are the future research and innovation directions to eventually translate our findings to real practice, with the final aim to innovate healthcare management with holistic, long term and comprehensive strategies.

## Additional files

Additional file 1: Detailed information on Lean Canvas, Focus Groups and Use Cases. (DOCX 20 kb)

Additional file 2: AHP Questionnaire - Solution 1. (DOCX 836 kb)

Additional file 3: AHP Questionnaire - Solution 2.1. (DOCX 836 kb)

Additional file 4: AHP Questionnaire - Solution 2.2. (DOCX 836 kb)

## Data Availability

The datasets used and/or analysed during the current study are available from the corresponding author on reasonable request.

## Abbreviations

AHP: Analytic Hierarchic Process
BMI: Body mass index
CDSS: Clinical Decision Support Systems
CGM: Continuous Glucose Monitoring
DSS: Decision Support Systems
EHR: Electronic health record
GP: General practitioner
HC: Healthcare
HCI: Human Computer Interaction
HE: Heuristic Evaluation
IT: Information Technology
MOSAIC: MOdels and Simulation techniques for discovering dIAbetes influence faCtors
NHS: National Health Service
SUS: System Usability Scale
T2D: Type 2 Diabetes
UC: Use case
UCs: Use cases
VC: Validation Case
WHO: World Health Organization
