# Supplementary material for: What do healthcare professionals need to turn risk models for type 2 diabetes into usable computerized clinical decision support systems? Lessons learned from the MOSAIC project
Source: BMC Med Inform Decis Mak. 2019 Aug 16;19:163. doi: 10.1186/s12911-019-0887-8 (PMC6697904; doi:10.1186/s12911-019-0887-8)
Supplement: Supplementary file 1 — Detailed information on Lean Canvas, Focus Groups and Use Cases. (DOCX 20 kb) [file 12911_2019_887_MOESM1_ESM.docx]

# Appendix Detailed information on Lean Canvas, Focus Groups and Use Cases

Table 1 – Definition of the Problem, Objectives and Target Customers using Lean Canvas

| Background | *The interest in identifying people at risk of developing T2D gained extreme importance because of the pandemic dimension reached by the disease. The WHO estimates by 2030 there will be about 550 million people suffering T2D. The current diagnostic criterion is focused on identifying groups with significantly increased prevalence of microvascular complications. This suggests that the current diagnostic methods are missing the opportunity to identify early symptoms of T2DM, which leads to later detection and treatment of patients and the consequent development of complications, which could be avoided with an earlier intervention.* |
| --- | --- |
| The problem | *To respond to the unmet need of improving T2D diagnosis and management: how can computer modelling techniques make a positive impact to this need?* |
| Objectives | *To solve “the problem”, two main objectives have been agreed by the experts:*   1. *To use physiological and probabilistic modelling to discover environmental and clinical risk factors associated to the onset of T2D* 2. *To use data mining techniques to predict the development of complications and assess the evolution of the disease*   *According to the existing healthcare systems, reliable information on detection and prediction of diabetes can be obtained from clinical studies, while, once diabetes is diagnosed, information can be derived from health information systems.* |
| Target Customers | 1. *Healthcare Providers (Primary Care, Specialists, Nurses, Case Managers, etc.)* 2. *Healthcare Managers (Heads of Service, NHS´s Departments of Planning, Private Insurers, etc.)* 3. *Patients and Citizens (including community-based associations)* |
| Solution 1: improving detection and prevention of T2D | *T2D Screening: Through the combination of databases from different clinical studies, tools to model the metabolic evolution of individuals across the time, taking into account physical, metabolic, phenotypic and lifestyle variables can be developed.*  *This will allow for early diagnosis of Type 2 Diabetes Mellitus, leading to control the evolution and to avoid or delay the onset of the illness when patients are shown to be already diabetic or to revert the metabolic impaired situation through lifestyle intervention in cases where patients belong to pre-diabetic states.* |
| Solution 2: improving care and follow up of T2D | 1. *T2D Management, to be used at a population level, helps Hospital and Health Care Agency Managers to understand, in their hospitals and regions, the evolution of the disease at population level and the use of resources. This tool has the potential to ease the strategic approach of the socioeconomic problem of T2DM as far as the information it provides will help to improve the internal organization of the healthcare system, estimate the clinical specialties which will have a higher workload in the future years, the differences between gender issues, among different regions, as well as measure, compare and analyze the impact of different approaches to diabetes care in different healthcare organizations.* 2. *Support during Follow-up visits, to be used at an individual level, aims to enhance the capability of the treating diabetes specialists and GPs to act as central point from which pivoting the coordination of the whole clinical and social specialties involved in the chronic management of T2D. Through computerized inclusion of clinical guidelines as well as the timetables, consultations and tests performed or “to perform” with an intelligent alert system we will enable the comparisons between the designed clinical pathways/flows and the real flows occurring in individual patients.* |

Table 2 – Detailed description of User Needs Gathered through the Thematic Focus Groups

| **Clinical Focus Group:**  *Solution 1: improving detection and prevention of T2D.* | Importance of excluding False Positives |
| --- | --- |
|  | Importance to reduce unnecessary workload in existing healthcare systems |
|  | Reluctance in discovering True Positives because this would cause additional costs and effort |
|  | The solution does not have to bring too high additional costs in the short-term, even if it demonstrates to be cost-effective in the long-term. |
|  | Connection with existing Health Information Systems, registries or records should represent a low-cost approach to explore. |
|  | The solution could be extremely useful if it demonstrates to reduce the workload of the nurses and support them in delivering lifestyle modification interventions and increase patients’ education. |
|  | The solution should help to improve existing screening strategies and help clinicians, policy makers and citizens to increase their awareness on the disease. |
| **Clinical Focus Group:**  *Solution 2: improving care and follow up of T2D* | Need to know the real status of the clinical unit, supporting data analysis, visualization of changes and providing the necessary knowledge about what is going on in the clinical unit. Supporting the release of reports and performing assessments. |
|  | To improve the time of visits, to identify differences between clinical guidelines and real pathway and to improve interactions among Head of Services, Nurses and Specialists. |
| **Scientific Focus Group** | Suggestion to avoid using subjective data for modeling purposes, as it is almost impossible to standardize this kind of data |
|  | Importance of using time to event for predictions, rather than fixed temporal horizons. |
|  | The analysis of medication-related parameters as predictors or proxy of complications is a novel and interesting research line to proceed |
|  | In the future, we should be able to diagnose diabetes through continuous glucose monitoring (CGM) of 48-72 hours. This should return the same results as HbA1c test, but without other cofounding variables (i.e. iron deficiency, glycation rate). CGM should be considered also to predict the appearance of complications. |
| **Business** | Importance to identify early adopters like Private Healthcare Providers, Innovation Unit of Public Healthcare Institutions or Public Organizations that manage social and healthcare services for public employees. |
|  | Interoperability, connectivity with existing Healthcare Information Systems and Electronic Health Records have to be addressed. |
|  | Insurance Companies are reluctant and scared about adoption of this kind of solutions. This is mainly due to: unavailability and prohibition to use patient data, annual-basis insurance discouraging prevention activities. |

Table 3 – The MOSAIC Solutions and Use Cases

| *Solution* | *Use Cases* | *Beneficiary* | *User* |
| --- | --- | --- | --- |
| *Solution 1: improving detection and prevention of T2D.* | **UC1.1: Risk Factors and Indicators to be adapted to Public Health**. Development of a tool for improving the characterization of early T2D. The tool will receive in input available information on the patient: independent variables (gender, age education, etc.); habits (smoking, etc.); phenotypic variables (cardiovascular events, etc.); metabolic variables (e.g. indices for modelling insulin resistance/sensitivity and β-cells function, etc.); and glucose variability indices extracted from continuous glucose monitoring traces. The output of the tool will be a probability of belonging to a specific class (low, medium and high risk of T2D). | National/ regional/local healthcare agencies | Diabetologist |
|  | **UC1.2: Risk Factors and Indicators to be adapted into ambulatory settings.** The same tool of UC1.1 can be used in Ambulatory settings where screening can be done on the treated population and people at risk of developing T2D could be screened and action taken on an individual level. | Primary Care (private or public) | GP and specialists, depending on the case |
|  | **UC1.3: Risk Factors and Indicators to be adapted to citizens for personal use.** In this case the tool can be integrated in a system to be used by citizens directly, to assess their risks of developing T2D | NHS, Private Care Organization and Citizens | Citizens |
| *Solution 2: improving care and follow up of T2D* | **U2.1: Top-down analysis for decision makers** Starting from patients' groups, clustered on the basis of their risk of complications, identify subgroups with similar health care trajectories. Detect the most critical pathways, in terms of severity and use of resources, and plan suitable clinical and organizational actions. | Primary Care (private or public), Private/Public Hospitals | Clinician treating Diabetic Population |
|  | **UC2.2: Decision support for clinicians** Development of new strategies for stratifying patients treated in a specific clinical context on the basis of their temporal clinical history. Each patient will be assigned to a reference multivariate temporal pattern extracted at the hospital population level. Assess the risk of developing complications within the groups characterized by similar temporal patterns. | Primary Care (private or public), Private/Public Hospitals | Healthcare Managers, Head of division |
