# Supplementary material for: What do healthcare professionals need to turn risk models for type 2 diabetes into usable computerized clinical decision support systems? Lessons learned from the MOSAIC project
Source: BMC Med Inform Decis Mak. 2019 Aug 16;19:163. doi: 10.1186/s12911-019-0887-8 (PMC6697904; doi:10.1186/s12911-019-0887-8)
Supplement: Supplementary file 4 — AHP Questionnaire - Solution 2.2. (DOCX 836 kb) [file 12911_2019_887_MOESM4_ESM.docx]

| 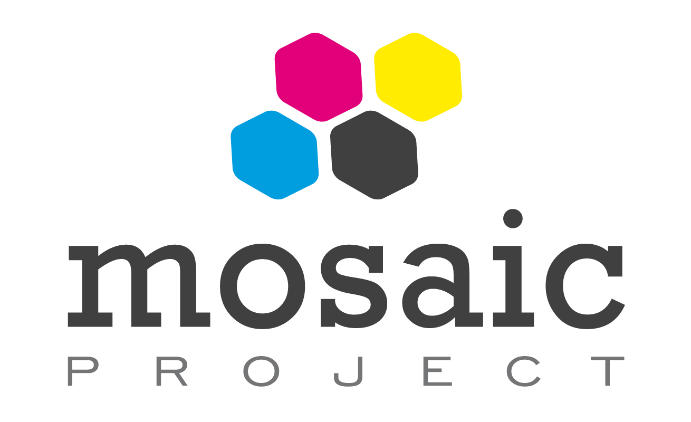 | 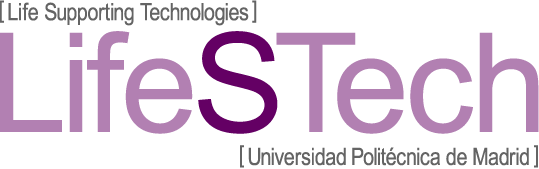  Universidad Politecnica de Madrid  Avd. Complutense n30  28040 Madrid, Spain  www.lst.tfo.upm.es |
| --- | --- |

User Needs for incorporating diabetes modeling techniques in disease management tools, using the Analytic Hierarchy Process (AHP)

First of all, thank you for your time and for taking part of this study aimed at evaluating the key aspects of which are the most important factors to take into account when developing tools for assisting Medical Doctors (MD) during visits with T2DM patients.

## Introduction

Type 2 Diabetes Mellitus (T2DM) is the most common form of diabetes. It accounts for at least 90% of all cases of diabetes. The World Health Organization (WHO) estimates that by 2030 there will be about 550 million people suffering this disease. This disease can remain undetected for many years because hyperglycemia (consequence of the insulin defects) develops gradually and at earlier stages is not severe enough for the patient to notice any of the classic symptoms of diabetes.

Despite the large number of models being developed and the increased interest and acknowledgement in the clinical field, only a very small minority ends up being used in the clinical practice.

One of the most important challenges of the MOSAIC project is to combine the research activities related to the discovery of new risk factors, methods and models for diabetes onset, progression and evolution, with the development of software tools, components and modules that would incorporate such innovations and make them usable by different end-users in a variety of settings and purposes. In other words, the challenge consists in let innovations achieving impact in the current clinical practice.

For this study, we have identified outcomes and then created an initial hierarchy of needs that can be used by developers and human computer interaction experts in the system design.

## Consent form

You have been selected to be part of this study as expert in the field. The data collected will be associated to your profile and background in order to analyze your opinion as expert but your name or any other personal detail will not be disclosed at any time. Your opinion will only be used for research purposes. By taking part of this study and submitting your response you will agree with these conditions.

## Objective

The main objective is to understand, through your help, which are the real needs and challenges in the development and deployment of novel technological solutions for assisting MD during individual visits with T2DM patients. For each pairwise comparison you should always follow this criteria:

“In a technological solution that support you in understanding the clinical evolution of your patient, and from your point of view and according to your experience, which is the most important element and how much important it is, with respect to the other one (from 1, equally important to 9 much more important)?”

## The Analytic Hierarchy Process (AHP)

The analytic hierarchy process (AHP) is a structured technique for organizing and analysing complex decisions or systems. It was developed by Thomas L. Saaty in the 1970s and has been extensively studied and refined since then and applied in a wide variety of decision situations, in fields such as government, business, industry, healthcare, and education. Rather than prescribing a "correct" version, the AHP helps decision makers find one that best suits their goal and their understanding of the problem. In this context the AHP has been proposed to elicit user needs (Pecchia et al. 2013) and to assess the medical technologies in their early stages. The complexities of medical device decision making require to collect and organise a spectrum of qualitative and quantitative information in a systematic way (Pecchia et al. 2013). Users of the AHP first decompose their decision problem into a hierarchy of more easily comprehended sub-problems, each of which can be analysed independently. And, once the hierarchy is built, experts in the field can use it to systematically evaluate its various elements by comparing them to one another two at a time, with respect to their impact on an element above them in the hierarchy. The AHP converts these evaluations to numerical values that can be processed and compared over the entire range of the problem. This hierarchical approach allows the construction of a consistent framework for step-by-step decision-making, breaking a complex problem into many small less-complex ones that decision-makers can more easily deal with (Pecchia et al. 2013).

## How this works

Basically, and to sum up the methodology, AHP breaks down complex system in “smaller parts” and then asks an expert group to compare and rate these elements in several pairwise comparisons. The mathematics behind AHP allows us to calculate the “Consistency ratio” which measures how coherent are your responses. To illustrate what this, take a look at the following example, let’s say that you state:

(Criteria A > Criteria B) AND (Criteria B > Criteria D)

Then Criteria A >> Criteria D, nevertheless, as we said at the beginning we are analysing complex systems and consequently it is very difficult to rank properly all the Criteria. So, it is possible and common that later in the evaluation the user states that Criteria A > Criteria D or even Criteria A < Criteria D. In this case, and thanks to the AHP method we can quantify the inconsistencies (usually values under 10% are accepted) and suggest the evaluator to modify some decisions to improve the consistency.

## The hierarchy

The first step in AHP is the definition of the criteria or hierarchy that you as expert should evaluate. In order to identify elements of the hierarchy a literature review was performed and then a focus group with experts was consulted to organize these factors. The focus group identified a total of 19 factors (Fico et al. 2014). Then, they were redefined in order to group all the common needs and to avoid misunderstandings, repetitions and overlapping. The table below shows the final criteria list.


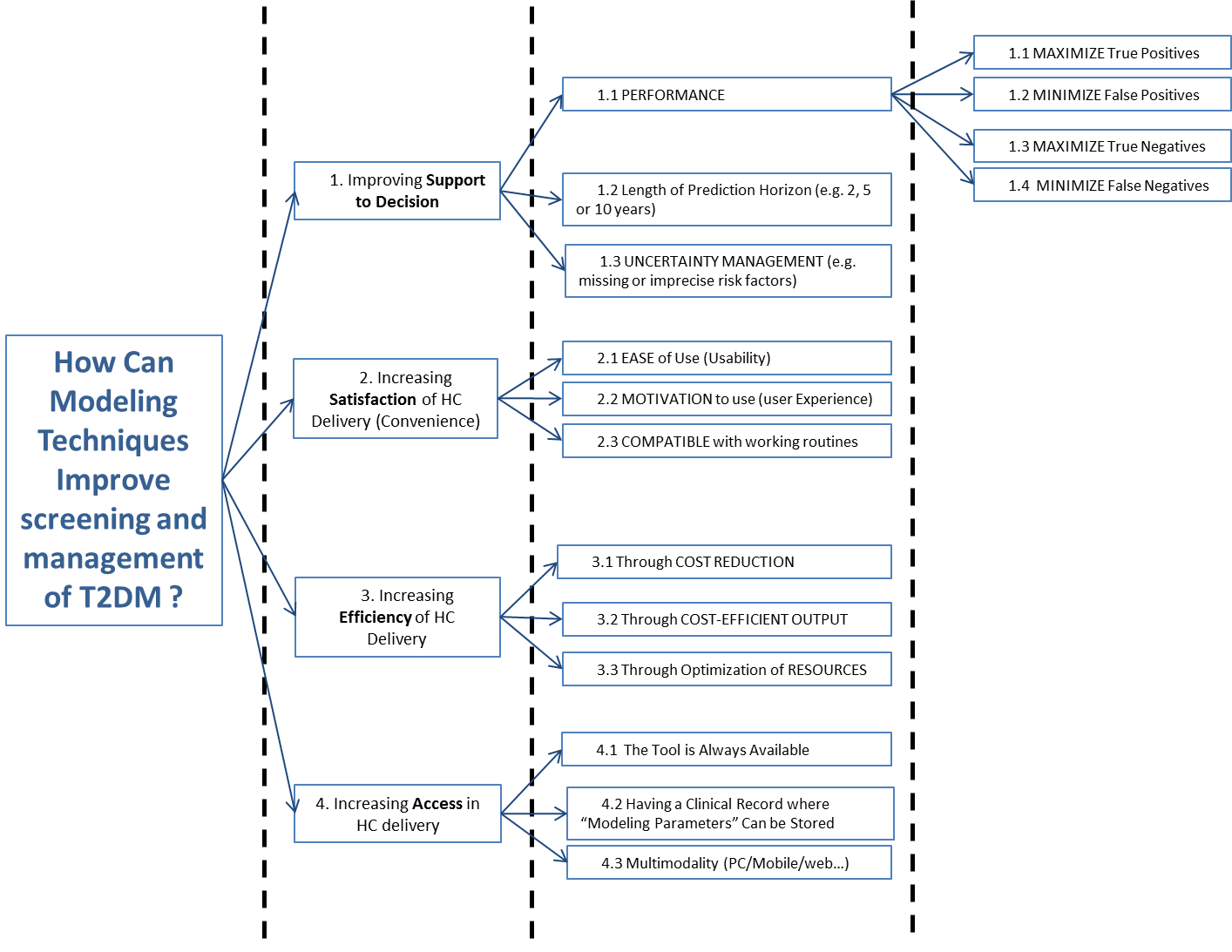


## Step by Step guide

Now, to take the test click on the following link or Copy&Paste this link in the navigation bar of your web browser:

<http://bpmsg.com/academic/ahp-hiergini.php?sc=rYvEzu&pn>

1. The first screen show a Session Code and personal identifier in the field Your Name. In the case these fields are empty please introduce the following details.

- Session code: rYvEzu
- Your name:

Click “Check input” button, after that, click on the “Go” button.


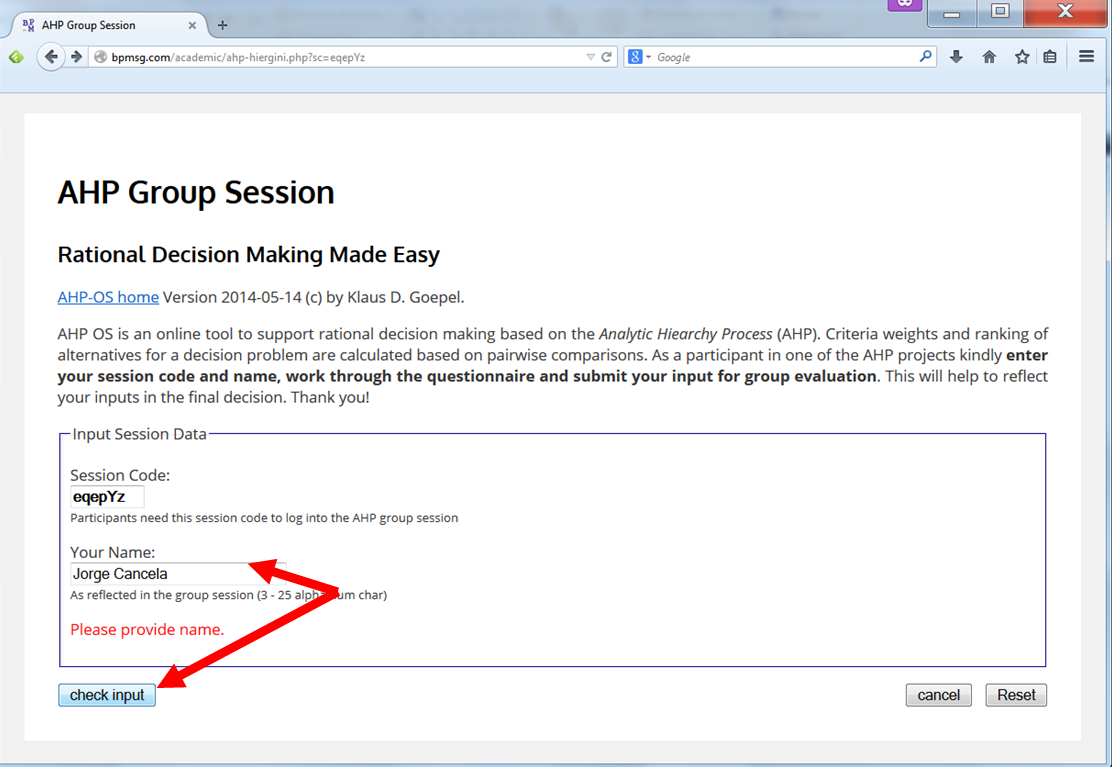


1. At this point the tool shows you the hierarchy, organized in 6 nodes and 20 elements within these nodes. From here you should rate the elements in the nodes, and also the nodes themselves. To do that, click on the “AHP” buttons (in total you should carry out 6 evaluations).

IMPORTANT: Please click on the 1^st^ AHP button on the right column, and perform the corresponding comparisons, then on the 4 AHP buttons of the central column before clicking the final and 5^th^ one, which compares the 4 nodes.


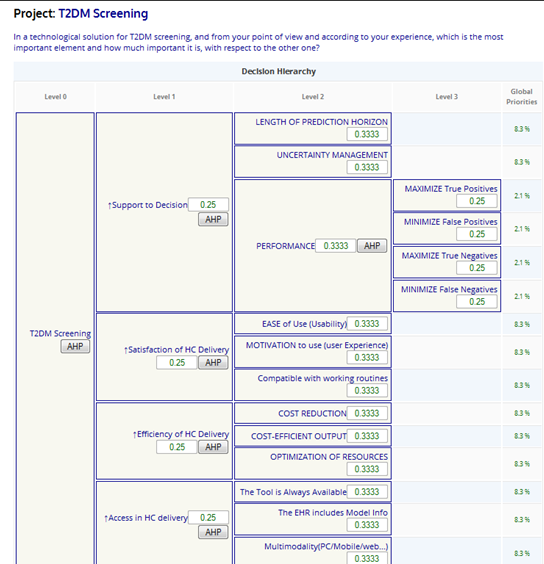


1. The procedure is the same in all the cases

Each row shows you a pairwise comparison that you should evaluate.

You have to choose which one of the two criteria showed per row is more important and then rate it from 1 (equal important) to 9 (much more important).

“In a technological solution that support you in understanding the clinical evolution of your patient, and from your point of view and according to your experience, which is the most important element and how much important it is, with respect to the other one (from 1, equally important to 9 much more important)?”

1. When you finish the evaluation of all the pairwise comparisons click on the “Calculate Result” button and it will evaluate the Consistency Rate and it will show you the result.

- If the Consistency Rate is higher than 10% the tool will advise you that the rate is too high and even it will suggest you with some possible modification that can lead to improve the CR.
- To fix it, this tool shows you some recommendations to improve the consistency.
- It will highlight in green its suggestions.
- Of course, this is only a recommendation, taking into account the valuation of the other pairwise comparisons. You are free to adjust the comparisons as you better estimate.


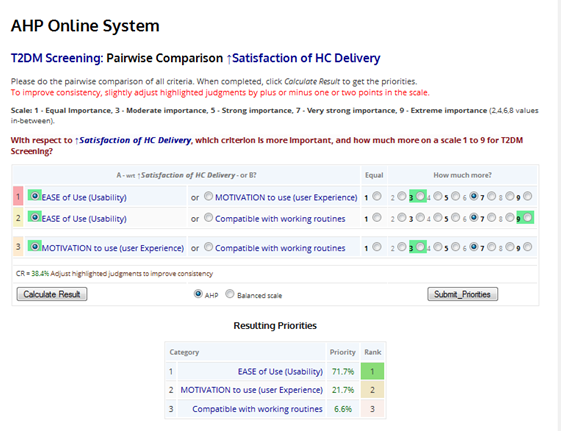


1. When the Consistency rate is under 10% click on the Submit_Priorities button.


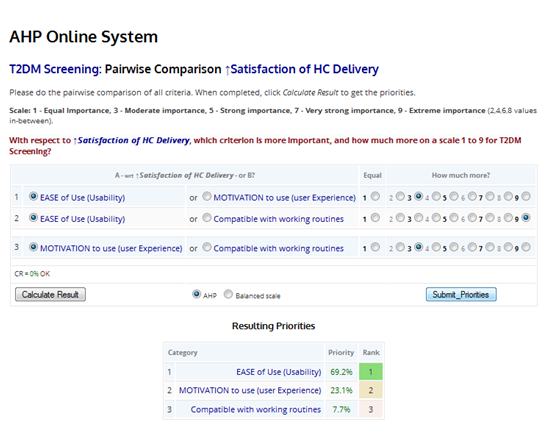


1. Repeat this action with all the AHP buttons and once you are done with all of them, click on the “Submit for group eval” button.


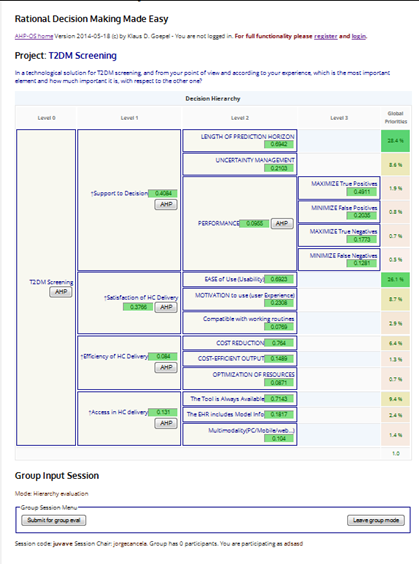


That’s all, thank you again for taking part of this study and for your time.

Best regards,

Giuseppe Fico

[gfico@lst.tfo.upm.es](mailto:gfico@lst.tfo.upm.es)
